# Involvement of p38 MAPK in Leydig cell aging and age-related decline in testosterone

**DOI:** 10.3389/fendo.2023.1088249

**Published:** 2023-03-06

**Authors:** Dandan Luo, Xiangyu Qi, Xiaoqin Xu, Leilei Yang, Chunxiao Yu, Qingbo Guan

**Affiliations:** ^1^ Department of Endocrinology, Shandong Provincial Hospital Affiliated to Shandong First Medical University, Shandong University, Jinan, Shandong, China; ^2^ Shandong Provincial Key Laboratory of Endocrinology and Lipid Metabolism, Institute of Endocrinology and Metabolism, Shandong Academy of Clinical Medicine, Jinan, Shandong, China

**Keywords:** Leydig cells, testosterone, p38 MAPK, aging, obesity

## Abstract

**Introduction:**

Age-related decline in testosterone is associated with Leydig cell aging with impaired testosterone synthesis in aging. Obesity accelerates the age-related decline in testosterone. However, the mechanisms underlying the Leydig cell aging and the effects of obesity on Leydig cell aging remain unclear.

**Method:**

Natural aging mice and diet-induced obese mice were used to assess the process of testicular Leydig cell senescence with age or obesity. Bioinformatic analysis of the young and aged human testes was used to explore key genes related Leydig cell aging. Leydig cell-specific p38 MAPK knockout (p38LCKO) mice were used to further analyze the roles of p38 MAPK in Leydig cell aging. The levels of testosterone and steroidogenic enzymes, activity of p38 MAPK, aging status of Leydig cells, and oxidative stress and inflammation of testes or Leydig cells were detected by ELISA, immunoblotting, immunofluorescence, and senescence-associated β-galactosidase (SA-β-Gal) staining analysis, respectively.

**Result:**

The serum testosterone level was significantly reduced in aged mice compared with young mice. In the testis of aged mice, the reduced mRNA and protein levels of LHCGR, SRB1, StAR, CYP11A1, and CYP17A1 and the elevated oxidative stress and inflammation were observed. KEGG analysis showed that MAPK pathway was changed in aged Leydig cells, and immunoblotting displayed that p38 MAPK was activated in aged Leydig cells. The intensity of SA-β-Gal staining on Leydig cells and the number of p21-postive Leydig cells in aged mice were more than those of young mice. Similar to aged mice, the testosterone-related indexes decreased, and the age-related indexes increased in the testicular Leydig cells of high fat diet (HFD) mice. Aged p38LCKO mice had higher levels of testosterone and steroidogenic enzymes than those of age-matched wild-type (WT) littermates, with reduced the intensity of SA-β-Gal staining and the expression of p21 protein.

**Conclusion:**

Our study suggested that obesity was an important risk factor for Leydig cell aging. p38 MAPK was involved in Leydig cell aging induced by age and obesity. The inhibition of p38 MAPK could delay Leydig cell aging and alleviate decline in testosterone.

## 1 Introduction

In males, serum testosterone decreases with age after the age 40 years, known as age-related decline in testosterone (1). Decreased testosterone causes infertility, sexual dysfunction, and age-related symptoms in older male populations known as late-onset hypogonadism (2). Population aging remarkably increases the incidence of this disease (3). Men with testosterone deficiency benefit a lot from testosterone replacement therapy (TRT), including improved bone density and strength, body composition, sexual function, and mood (4, 5). However, the long-term effects of TRT on major cardiovascular events and prostate cancer risk in older men remain controversial. Recent clinical trials in older men have shown inconsistent effects of TRT on cardiovascular events, with some showing positive effects, a reduced risk of atrial fibrillation and myocardial infarction (6–8), some showing negative effects, an increased the risk of death, myocardial infarction, and stroke (9–11), and others showing no effects (12). And the controversial observations also exist in the effects of TRT on prostate cancer (13–16). The reason for these results might be related to the subject characteristics and the length of treatment. Neither the clinical benefits nor the long-term safety of TRT has been fully established in older men with age-related decline in testosterone, thus, elucidating the risk factors and molecular mechanisms of age-related decline in testosterone will contribute to the prevention and treatment of this disease.

Obesity is considered a major culprit of cell aging and aging-related diseases, such as neurodegenerative disorders, cardiovascular diseases, and cancer (17–19). Cellular alterations caused by obesity, such as oxidative stress, inflammation, and DNA damage accumulation are the potential driving factors for the aging process (20). Epidemiologic studies show that obese men have lower testosterone compared to lean men in diverse ethnic population (21–24). Recent studies suggest that obesity is related to age-related decline in testosterone. In the European male aging study, obesity is the greatest determinant of the variance in serum testosterone with age (25). Serum testosterone decline with age in obese patients is more pronounced than in lean individuals (26). Weight control in obese older men is associated with an increase in total testosterone and free testosterone (27). All the above studies suggest that obesity epidemic has the potential to exacerbate the age-related decline in testosterone.

Serum testosterone in males is synthesized by testicular Leydig cells under the control of luteinizing hormone (LH). Steroidogenic acute regulatory protein (StAR) and a series of steroidogenic enzymes (CYP11A1, 3β-HSD, and CYP17A1) are responsible for testosterone synthesis. Accumulating evidence suggest that age- or obesity-related decline in testosterone is correlated with impaired testosterone biosynthesis of Leydig cells. The level of LH is unchanged or slightly increased in most males (28, 29). Decreased expressions of StAR and steroidogenic enzymes account for impaired function of Leydig cells (27, 30, 31). Whether the impaired function of Leydig cells in aged males follows cell aging, and whether the obesity accelerates Leydig cell aging remains unknown. Few works have been done to investigate the Leydig cell aging and the underly mechanisms.

The p38 mitogen-activated protein kinase (p38 MAPK) belongs to the family of MAPKs, which is involved in a variety of cell stress response (3). Oxidative stress and chronic inflammatory state, the common pathological features underlying aging and obesity, are strong inducer of p38 MAPK activation (32). It has been demonstrated that p38 MAPK is implicated in the obesity- and age-related diseases (33, 34). Recent reports showed that p38 MAPK is activated in Leydig cells under oxidative stress and might be involved in reduced expression of StAR (35). Whether the p38 MAPK is involved in the Leydig cell aging with age or obesity in still unknow.

In the present study, we found that obesity was a risk factor for Leydig cell aging. p38 MAPK was implicated in Leydig cell aging and age- and obesity-related decline in testosterone.

## 2 Materials and methods

### 2.1 Materials

Testosterone and LH enzyme-linked immunosorbent assay (ELISA) kit was obtained from CUSABIO (Wuhan, China). Mouse TNFα, IL-1β, and IL-6 ELISA were obtained from Lianke Biotech (Hangzhou, China). SOD and MDA detection kit and senescence β-galactosidase (SA-β-gal) staining kit were purchased from Biyuntian (Shanghai, China). The bicinchoninic acid (BCA) protein assay kit was purchased from Shenneng Bocai (Shanghai, China). RNAiso reagent and reverse transcription kit were purchased from Takara (Tokyo, Japan). SYBR green PCR master mix was purchased from Yeasen (Shanghai, China). Antibodies against phosphorylated p38α MAPK (p-p38 MAPK, Thr180/Tyr182), p38α MAPK, phospho-extracellular signal-regulated kinase 1/2 (anti-p-ERK1/2, Thr202/Tyr204), anti-ERK, anti-p-c-Jun N-terminal kinase (p-JNK, hr183/Tyr185), anti-JNK, StAR and CYP11A1 were purchased from CST (Boston, MA, USA). Antibodies against CYP17A1, Hsp90, and Tubulin were from Proteintech (Wuhan, China). Mouse monoclonal antibodies against p21 and p16 were purchased from Santa Cruz Biotechnology (Santa Cruz, CA, USA). Antibody against p-p38 MAPK (Thr180) for IHC was obtained from ZEN-BIOSCIENCE (Chengdu, China). All other chemicals used in this study were of analytical grade and obtained from Sigma (St. Louis, MO, USA) unless otherwise stated.

### 2.2 Animals

The experimental design and animal treatment protocol were approved by the Animal Ethics Committee of Shandong Provincial Hospital. Animals were raised in controlled environmental conditions (22 ± 2°C; 12h light/dark cycle) with food and water ad libitum.

(1) 3, 6, and 18 month of age C57/BL6 mice were purchased from Beijing Weitong Lihua Biotechnology Co., Ltd.
(2) 8 weeks male C57/BL6 mice were purchased from Beijing Weitong Lihua Biotechnology Co., Ltd. Mice were randomly allocated into two groups. Normal diet group (ND) mice received the diet containing only 11.6% kcal fat, 23% kcal protein and 65.4% kcal carbohydrate (Keaoxieli, China). High fat diet (HFD) mice received the diet providing 60% kcal fat, 20% kcal protein and 20% kcal carbohydrate (D12492 Research Diets, USA) for 24 weeks.
(3) Leydig cell-specific p38α knockout was generated by mating p38α^flox/flox^ (p38α^f/f^) mice to Cyp17a1-iCre mice. The transgenic mouse line Cyp17a1-iCre, in which an enhanced form of Cre recombinase (iCre) gene expression is driven by a promoter of 17β-hydroxylase α1 (36). The Cyp17a1-iCre mice were purchased from Jackson Laboratory (Bar Harbor, ME, USA). Cre-mediated recombination was visualized by using of a reporter mouse strain with the Cre inducible tdTomato-reporter (ROSA26Sor-lox-STOP-TdTomato). This reporter mouse was from model animal research center of Nanjing university (Nanjing, China).The floxed p38α mice, contains loxP sites in the introns flanking exon 1 of the p38α gene (37) were the generous gift from the University of Chinese Academy of Sciences. The p38α^f/f^ mice with iCre are p38LCKO mice, while p38α^f/f^ mice that do not express iCre served as WT controls. Primers for PCR genotyping of Cyp17a1-iCre F: GGA CTC AGG CTT GGA GAC ACT T; R: AGG TGC TGT TGG ATG GTC TTC A; Primers for PCR genotyping of floxed p38α F: TCC TAC GAG CGT CGG CAA GGT G; R: GTC CCC GAG AGT TCC TGC CTC.

The mice were sacrificed at different times according to the experimental plan. Serum samples were collected from eyeball blood in anesthetized mice. Blood samples centrifuged at 3000rpm for 15 min and stored at −80°C until assayed. One testis of each mouse was stored at −80°C for subsequent experiments. Another one was fixed in mDF (37–40% formaldehyde, absolute ethanol, glacial acetic acid, distilled water volume ratio of 3:1.5:0.5:5) for 24h for subsequent analysis.

### 2.3 Assessment of serum lipid levels

To evaluate the effect of HFD on serum lipid in mice, serum total cholesterol (TC), triglyceride (TG), high-density lipoprotein-cholesterol (HDL-C) and low-density lipoprotein-cholesterol (LDL-C) were measured directly using automatic biochemical analyzer (Beckmen, AU5831, USA) at the clinical laboratory of Shandong Provincial Hospital.

### 2.4 Assessment of serum hormone and testicular testosterone levels

The mouse serum testosterone and LH levels were measured by an ELISA kit according to the manufacturer’s protocols. For intratesticular testosterone assay, testis tissues (10 mg) were homogenized by a tissue homogenizer in 100 μl phosphate buffered solution. Then the homogenates were lysed by three times freeze-thaw and centrifuged at 12,000 rpm for 10 min to obtain the supernatant. Testosterone concentrations were detected with ELISA kit for testosterone according to the kit was expressed as ng/mg tissue weight

### 2.5 Detection of antioxidant enzymes in testis tissue

Testis tissues (10 mg) was homogenized with 100μl assay buffer and centrifuged at 12,000 rpm for 10 min to obtain the supernatant. Then the contents of MDA and SOD were obtained by using respective assay kit according to the manufacture’s protocols. Then they were normalized to protein concentrations, which is examined by BCA protein assay kit.

### 2.6 Detection of inflammatory factors in testis tissue

The testis tissue was also prepared for inflammation factors analysis by repeated freeze-thaw treatments. The levels of TNFα, IL-1β, and IL-6 were detected with ELISA kits according to the kit manufacturer’s protocol and then were normalized to protein concentrations.

### 2.7 Senescence-associated β-galactosidase staining

SA-β-Gal staining of Leydig cells was performed utilizing a SA-β-Gal staining kit. The frozen sections (10μm) from mouse testis were fixed fixative solution and then incubated with fresh β-galactosidase staining solution at 37°C for at least 12 h. Aged cells displayed a blue color in the cytoplasm and the intensity of staining indicated the cell senescence status. Images were acquired using a light microscope (Imager A2, Zeiss, Germany).

### 2.8 Measurement of lipid content using oil red O staining

For examination of lipid content, the frozen sections (10μm) from mouse testis were fixed with 4% formalin for 30 min, washed with PBS, then stained with Oil Red O for 10 min. The nucleus was counterstained with hematoxylin. Images were acquired using a light microscope.

### 2.9 Single-cell sequencing analysis

Single-cell RNA sequencing data of young and old human testis (GSE182786) were downloaded from https://www.ncbi.nlm.nih.gov/geo/query/acc.cgi?acc=GSE182786. The count data was imported into the Seurat (version 4.1.1) R package for quality control. Genes detected in < 3 cells and cells where < 1000 genes had nonzero counts were excluded. The low-quality cells that had > 15% mitochondrial genes were also discarded. Library size normalization was performed in Seurat on the filtered matrix to obtain the normalized count. The count matrix for the respective gene in each sample were gained by using pseudobulks method. Then, we obtained the expression matrix and group information of the data. Subsequently, the differences between the two groups were analyzed by DESeq2 (version 1.34.0) R package.

### 2.10 RNA isolation and quantitative real-time PCR

RNAiso Reagent was used to extract total mRNA from testicular tissues. mRNA samples were reverse transcribed by reverse transcription kit according to the manufacturer’s instructions. The cDNA obtained by reverse transcription was used as a template for Quantitative real time PCR (Q‐PCR) conducted with the LightCycler480 (Roche). Use the 2−ΔΔCt method to calculate the relative expression of mRNA.

The sequences of the PCR primers (synthesized by Qingdaoqingke Biotech Co., Ltd., China) of Lhcgr (5′- GATGCACAGTGGCACCTTC and 5′- TCAGCGTGGCAACCAGTAG), Star (5′-GGAGCA GAGTGGTGTCATCAGA and 5′-AGGTGGTTGGCG AACTCTATCT), Cyp11a1 (5′-TGCTTGAGAGGCTGGAAGTTGA and 5′-CGG ATTGCGGAGCTGGAGAT), Cyp17a1 (5′-GTACCC AGGCGAAGAGAATAGA and 5′-GCCCAAGTCAAA GACACCTAAT), 3β-hsd (5′-AGCTCTGGACAAAGT ATTCCGA and 5′-GCCTCCAATAGGTTCTGGGT) and β-actin (5′-GGCTGTATTCCCCTCCATCG and 5′-CCA GTTGGTAACAATGCCATGT).

### 2.11 Immunoblotting analysis

Tissue extracts were prepared by protein lysis buffer (RIPA: PMSF = 100:1) with a phosphatase inhibitor cocktail, and concentration was subsequently determined. Protein sample (60µg) was separated by 10 or 12% separating gel and electrophoretically transferred to polyvinylidene difluoride membranes (PVDF membrane, Millipore Corporate). The membranes were blocked with 5% milk in PBS for 1 h and incubated with primary antibodies overnight at 4°C. The membranes were subsequently incubated with secondary antibodies. Finally, the bands were visualized by chemiluminescence and quantified by Photoshop (Adobe Software). The relative expression levels of tested proteins were normalized to the corresponding tubulin.

### 2.12 Hematoxylin and eosin staining

To verify the histopathological changes of the testis, testicular tissue samples were dehydrated in a graded series of ethanol solutions and embedded in paraffin. Sections (5 μm) were de-paraffinized, rehydrated, and histologically stained with hematoxylin-eosin (HE) according to standard protocols, then imaged with a light microscope.

### 2.13 Immunofluorescence and immunohistochemistry

For immunofluorescence (IF), fixed tissues were heat treated in Tris‐EDTA buffer for antigen retrieval. After washed by PBS, sections were blocked with 5% goat serum albumin, then incubated primary antibody overnight at 4°C. After that, sections were incubated with the FITC‐conjugated second antibody and DAPI for 1 h at room temperature. Each slice was observed with a confocal microscope (Leica, Germany).

For Immunohistochemistry (IHC), after antigen retrieval, the tissue sections were incubated with 3% peroxide blocking solution for 20 min at room temperature before adding primary antibody and were incubated overnight at 4°C. The slides were then labeled with horseradish peroxides-conjugated streptavidin. The chromogenic reaction that was developed using Liquid DAB Substrate according to the manufacturer’s instructions. Images were acquired using a light microscope.

### 2.14 Statistical analyses

Statistical analyses were conducted using GraphPad Prism (v.9, GraphPad Software Inc, CA). All samples were determined by an independent‐sample t test. All data were expressed as mean ± SD, and the difference was considered statistically significant if p< 0.05.

## 3 Results

### 3.1 A decline in serum testosterone and testosterone synthesis of aged male mice

**Figure 1:**
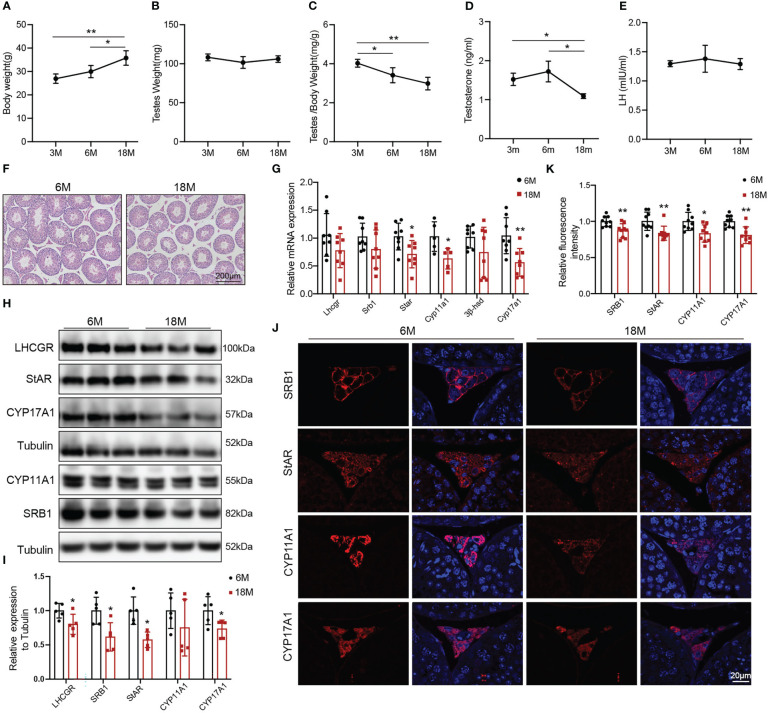
General features and testosterone synthesis of young and aged mice. **(A)** Body weight. **(B)** Testis weight. **(C)** Ratio of testis to body weight. **(D, E)** The levels of serum testosterone and serum luteinizing hormone (LH) were analyzed by ELISA. **(F)** Testis morphology was examined by HE staining. **(G)** The mRNA levels of proteins associated with testosterone synthesis were analyzed by Quantitative real time PCR. **(H)** Immunoblot assessed the protein levels of proteins related to testosterone synthesis. **(I)** Quantitative analyses for the protein level. **(J)** Immunofluorescence measured testosterone synthesis-related proteins. **(K)** Quantitative analyses of immunofluorescence. *p<0.05, **p<0.01.

The naturally aged mice were used to detect the age-related decline in serum testosterone and testosterone synthesis. We evaluated the general features and sex hormone levels of young mice (3 and 6 months) and aged mice (18 months). Body weight of aged mice was greater than that of 3-months old mice (**Figure 1A**, p<0.01) and 6-months old mice (**Figure 1A**, p<0.05). There was no change in mouse testis weight of all age (**Figure 1B**). The ratio of testis to body weight gradually decreased with age. The ratio of 3-months old mice was higher than that of 6-months old mice (**Figure 1C**, p<0.05) and 18-months old mice (**Figure 1C**, p<0.01). The serum testosterone level in aged mice was reduced compared with 3-and 6-months old mice (**Figure 1D**, p<0.05). There was no difference in LH level among three groups of mice (**Figure 1E**). Then, we selected 6-months old (young) and 18-months old (aged) mice to further evaluate the ability of Leydig cell to produce testosterone.

HE showed that the area of Leydig cells appeared normal across two groups of mice. Compared to the young mice, aged mice had lower height of the seminiferous epithelium (**Figure 1F**). The mRNA levels of the proteins associated with testosterone synthesis in Leydig cells of aged mice showed a decreasing trend, among which the difference in *Star* (p<0.05), *Cyp11a1* (p<0.05), and *Cyp17a1* (p<0.01) had statistically significances (**Figure 1G**). The protein levels of testosterone synthesis-related proteins were lower in aged mice than those in young mice (**Figures 1H, I**, p<0.05). The fluorescence intensity of SRB1, StAR, CYP11A1, and CYP17A1 were weaker in Leydig cells of aged mice than those in young mice with statistically significances (**Figures 1J, K**, p<0.05). The above results confirmed that the ability of Leydig cell to produce testosterone in aged mice was decreased.

### 3.2 p38 MAPK was activated in Leydig cells of aged mice

**Figure 2:**
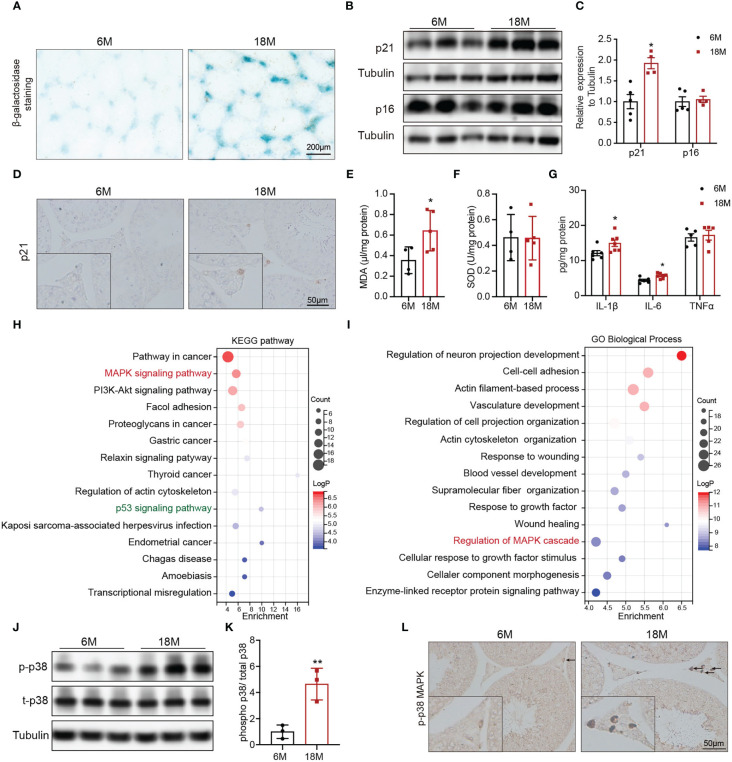
Age-related alterations in testes and Leydig cells. **(A)** Senescence-associated β-galactosidase staining in testes of young and aged mice. **(B)** The p21 and p16 protein expression in testis of two group mice were analyzed by immunoblot. **(C)** Quantitative analysis for the protein levels of p21 and p16. **(D)** The p21 expression and location was analyzed by immunohistochemistry. The levels of MDA **(E)**, SOD **(F)**, and inflammation factors **(G)** in the testis tissue of two groups of mice were detected by assay kits. The top 15 KEGG terms **(H)** and GO terms **(I)** enriched in the differentially expressed genes in Leydig cells from young and old human testis single-cell RNA sequencing were listed with p value and gene numbers. The p-p38 MAPK protein level in testis of two groups of mice were assessed by immunoblot **(J)** and IHC staining **(L)**. **(K)** Quantitative analysis for protein levels of p-p38 MAPK. *p< 0.05, **p< 0.01.

To understand the alterations in physiological state of testis or Leydig cells with age, we evaluated related indexes of aging status, oxidative stress, and inflammation. Accumulation of SA-β-Gal and increased levels of p21 and p16 are the most common markers of cell aging. Compared with young mice, SA-β-Gal staining was deeper in testicular interstitium of aged mice (**Figure 2A**). And the protein level of p21 was also higher in Leydig cells of aged mice than that of young mice, detected by immunoblot and IHC staining (**Figures 2B–D**, p<0.05). SOD and MDA are two indices of oxidative stress. The levels of MDA were elevated in testis of aged mice compared with young mice (**Figure 2E**, p<0.05), and there was no difference in SOD (**Figure 2F**). The expressions of pro-inflammatory (IL-6 and IL-1β) were significantly increased in testis of aged mice (**Figure 2G**, p<0.05). The above results suggested the existence of oxidative stress and inflammation in aged mouse testis.

To mine molecular mechanisms related to Leydig cell aging, we obtained and further enriched the differentially expressed genes (DEGs) between young and aged Leydig cells from single-cell RNA sequencing results of young and old human testes. The KEGG pathway analysis revealed the MAPK pathway as one of the most significantly affected pathway (**Figure 2H**). The MAPK pathway was also enriched by GO analysis (**Figure 2I**). In addition, KEGG analysis also identified a significant enrichment of genes in the p53 signaling pathway, which is important for p21 transcriptional activation (**Figure 2H**).

Then, MAPK family, including ERK1/2, JNK and p38 MAPK were evaluated in young and aged Leydig cells. Immunoblot results showed that the levels of p-p38 MAPK in testis of aged mice were higher than that in young mice with statistically significance (**Figures 2J, K**, p<0.01). Meanwhile, the protein levels of p-JNK and p-ERK1/2 were not changed (**Supplementary Figure 1**). IHC staining of p-p38 MAPK showed that increased level of p-p38 MAPK in aged Leydig cells (**Figure 2L**). The above results suggested that activiated-p38 MAPK was involved in Leydig cell aging.

### 3.3 p38 MAPK was involved in obesity induced Leydig cell aging

**Figure 3:**
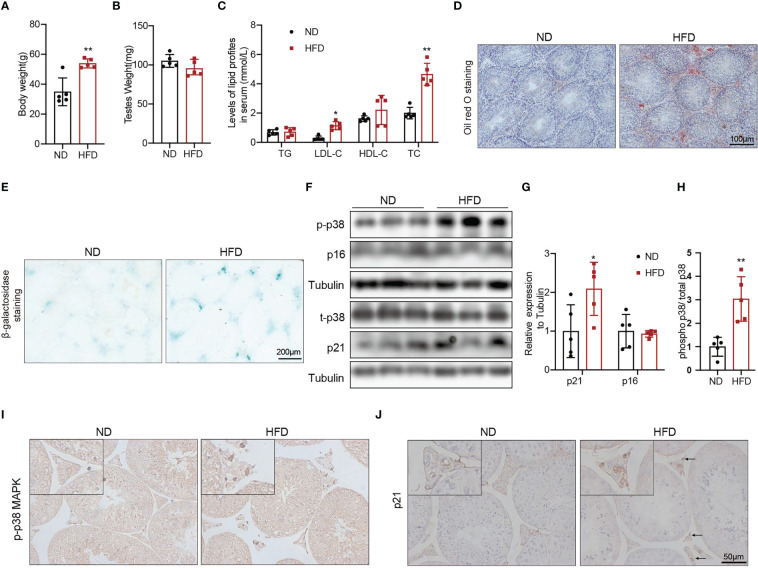
The aging status and p-p38 MAPK level of Leydig cells in mice fed with HFD or ND for 24 weeks. Body weight **(A)**, testis weight **(B)**, and serum lipid levels **(C)** were evaluated for mice. Lipid deposition in testicular tissues was detected by oil red O staining **(D)**. Aging status of Leydig cells was assessed by SA-β-Gal staining **(E)**. Immunoblot was used to detect the levels of p16, p21, and p-p38 MAPK **(F)**. Quantitative analysis for protein level **(G, H)**. IHC staining of p21 **(I)** and p-p38 MAPK **(J)** in testis tissue. *p<0.05, **p<0.01.

To explore whether obesity could accelerate the Leydig cell aging and the underly mechanisms, we used a high fat diet (HFD) to induce obesity in the mice. The 8-weeks male mice developed diet-induced obesity with the 60% high-fat diet for 24 weeks, as shown by increased body weight (**Figure 3A**, p<0.01). There was no difference in testis weight between the two groups of mice (**Figure 3B**). Serum LDL-C (p<0.05) and TC (p<0.01) were significantly elevated in mice fed with HFD compared with mice fed with ND (**Figure 3C**). The results of Oil Red O staining showed that obvious lipid deposited in the testis interstitium of mice fed with HFD, which was more than that of mice fed with ND (**Figure 3D**). The mRNA levels of the proteins associated with testosterone synthesis in Leydig cells of mice fed with HFD showed a decreasing trend. The protein levels of testosterone synthesis-related proteins, including SRB1, StAR, and CYP17A1 were lower in mice fed with HFD than those in control mice (**Supplementary Figure 2**, p<0.05).

We then evaluated the effects of β-galactosidase staining was deeper in testis interstitium of mice fed with HFD (**Figure 3E**). Immunoblot and IHC staining showed that the expression of p21 protein was higher in mice fed with HFD than that in mice fed with ND (**Figures 3F, G, J**), which indicated that obesity promoted Leydig cell premature aging. The levels of p-p38 MAPK detected by immunoblot and IHC staining in mice fed with HFD were higher compared with mice fed with ND (**Figures 3F, H, I**). All above results suggested that obesity was an important risk factor for Leydig cell aging, and p38 MAPK might be involved in obesity induced Leydig cell aging.

### 3.4 Leydig cell-specific p38 MAPK knockout alleviated age-related decline in testosterone

**Figure 4:**
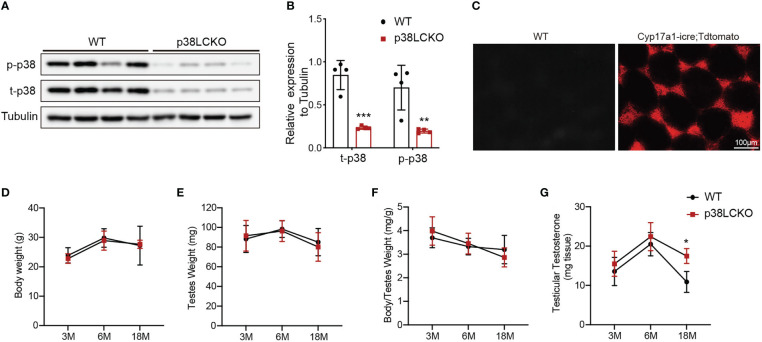
General features and serum testosterone levels of p38LCKO and WT mice. **(A)** The protein levels of t-p38 MAPK and p-p38 MAPK were analyzed by immunoblot. **(B)** Quantitative analyses for the protein levels. **(C)** Red fluorescence of Tdtomato in testis. The body weight **(D)**, testis weight **(E)**, ratio of testis to body weight **(F)**, and testicular testosterone **(G)** of p38LCKO and WT mic at 3-,6-, and 18- months of age. *p<0.05, **p<0.01, ***p<0.001.

We successfully generated Leydig cell-specific p38 MAPK knockout (p38LCKO) mice. As shown in **Figures 4A, B**, the levels of p-p38 MAPK and total p38 MAPK were decreased significantly in testis tissues of p38LCKO mice (p<0.01). Specificity of Cyp17a1-icre recombinase expression was determined by crossing with tdTomato reporter mice. Significant red fluorescence was only observed in the testis interstitium of Cyp17a1-iCre: tdTomato mice, while no fluorescence was observed in WT mice (**Figure 4C**).

Then we evaluated the features of p38LCKO mice at 3, 6, and 18 months of age. Body weight of p38LCKO mice at different ages were not different from those of age matched WT littermates (**Figure 4D**). Similarly, there were no differences in testis weight and the ratio of testis to body weight (**Figures 4E, F**) between two groups of mice. The above results illustrated that specific p38 MAPK knockout in Leydig cells did not affect the general features of mice.

There were no differences in serum and testicular testosterone level between two groups of mice at 3 and 6 months of age. At 18 months of age, serum and testicular testosterone levels were higher in p38LCKO mice than that of WT mice (**Figure 4G**; **Supplementary Figure 3A**, p<0.05). These results suggested that specific p38 MAPK knockout in Leydig cells could alleviate age-related decline in testosterone.

### 3.5 Specific p38 MAPK knockout improved the testosterone synthesis in aged Leydig cells

**Figure 5:**
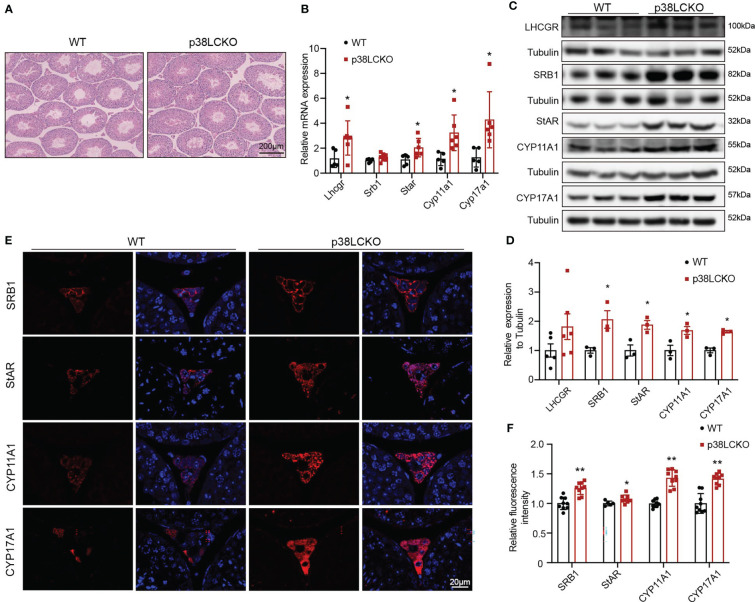
Assessment of ability of Leydig cell to produce testosterone in p38LCKO mice and WT mice at 18-month of age. **(A)** Testis morphology was examined by HE staining. **(B)** The mRNA levels of proteins related to were assessed by Q-PCR. **(C)** The protein levels of proteins associated with testosterone synthesis were analyzed by immunoblot. **(D)** Quantitative analysis for the protein level. **(E)** IF assessed the levels of testosterone synthesis-related proteins. **(F)** Quantitative analysis of fluorescence intensity. *p<0.05, **p<0.01.

The effects of Leydig cell-specific p38 MAPK knockout on steroidogenesis synthesis in aged mice were further assessed. The morphology of testis did not differ between both groups of mice at 3-and 6-month of age (**Supplementary Figure 3B**) and at 18-month of age (**Figure 5A**). The mRNA levels of *Lhcgr*, *Star*, *Cyp11a1*, and *Cyp17a1* were higher in 18-month-old p38LCKO mice than those in WT mice (**Figure 5B**, p<0.05). The levels of these proteins in Leydig cells were higher in p38LCKO mice at 18-month of age (**Figures 5C, D**, p<0.05). There were no differences in the mRNA and protein levels of those proteins in mice at age of 3 and 6 months between WT and p38LCKO mice (**Supplementary Figures 3C–F**). IF showed that, compared with aged WT mice, fluorescence intensity of the SRB1, StAR, CYP11A1, and CYP17A1 were stronger in p38LCKO mice (**Figures 5E, F**, p<0.05).

### 3.6 Specific p38 MAPK knockout delayed Leydig cell aging in aged mice

**Figure 6:**
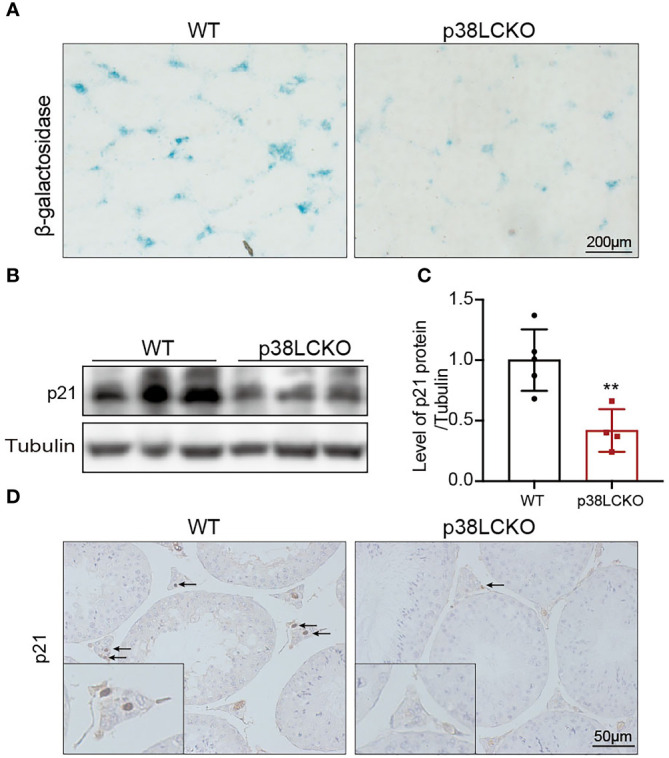
Assessment aging status of Leydig cell in p38LCKO mice and WT mice at 18-month of age. **(A)** SA-β-Gal staining in testes of two groups of mice was assessed by using staining kit. **(B)** The protein levels of p21 and p16 were detected by immunoblot. **(C)** Quantitative analysis for protein levels of p21 and p16. **(D)** IHC staining of p21 in testis tissue. **p<0.01.

To determine whether the Leydig cell-specific p38 MAPK knockout could delay cell aging, we assess the aging status of Leydig cell in p38LCKO mice at 18-month of age. Compared with WT mice, there was a weaker staining of SA-β-Gal in the testicular interstitium of p38LCKO mice (**Figure 6A**). The protein level of p21 was lower in p38LCKO mice than that in WT mice (**Figures 6B, C**). IHC staining of p21 showed that the number of p21-positive Leydig cells in p38LCKO mice was less than that in WT mice (**Figure 6D**). The above results showed that Leydig cell-specific p38 MAPK knockout could delay cell aging.

## 4 Discussion

Age-related decline in testosterone leads to testosterone deficiency in males, which can seriously affect male fertility and quality of life (1, 2). Elucidating mechanisms of aging and developing interventions are vital to reduced age-associated damage and functional decline. Aging is an irreversible natural process in human life which is influenced by many exogenous factors, such as living environment and diseases (38). Obesity is closely related to aging and accelerates the development of age-related diseases (20). It is unclear whether obesity promotes age-related decline in testosterone *via* accelerating Leydig cell aging. In this study, we demonstrated that obesity was an important factor to promote Leydig cell aging. p38 MAPK was involved in Leydig cell aging and age- and obesity-related decline in testosterone.

In present study, we found reduced serum testosterone in 18-month-old mice, illustrating a suitable model to explore the mechanisms under age-related decline in testosterone. In the adult testis, Leydig cell testosterone production depends upon the pulsatile secretion of LH by binding to LHCGR to maintain of optimal levels of steroidogenic enzymes and to mobilize and transport of cholesterol into the inner mitochondrial membrane *via* StAR. In this study, we found that the expressions of nearly all proteins associated testosterone synthesis were reduced, without changes in LH levels in aged mice. The above results were consistent with previous studies that age-related changes in Leydig cell steroidogenesis occurred at the gonadal level rather than secondary to hypothalamic-pituitary changes (39).

Despite cellular functional decline is the characteristic of cell aging, the aging status of Leydig cell in aged males is not directly evaluated. In this study, cell senescence markers, represented as expression of p21 and p16 protein and staining for SA-β-Gal were evaluated. The SA-β-Gal staining was present in testicular interstitium in aged mice. The level of testicular p21 protein was increased, and IHC showed that increased p21 focused on Leydig cells in aged mice. The above results suggested that Leydig cells were the prime target for cell aging in testes. And Leydig cell senescence was accompanied by resultant reduced testosterone synthesis.

To explore molecular related to Leydig cell aging, we obtained and further enriched the DEGs between Leydig cells from single-cell RNA sequencing results of young and old human testes. Both KEGG and GO terms enriched in MAPK pathway, a family of serine/threonine kinases consisted of three family members: JNK, ERK1/2, and p38 MAPK (32). In the present study, p38 MAPK, rather than JNK, ERK1/2 was activated in aged Leydig cells.

The JNK and ERK1/2, together with p38 MAPK belong to the MAPK family. ERK1/2 is activated by growth factors and cytokines and plays a central role in the control of cell proliferation and differentiation. In our study, there was no change in the expression of the p-ERK1/2 in testis of aged mice, which was compatible with previous perspectives of ERK1/2. The JNK and p38 MAPK have similar function and are known as stress-activated protein kinase and are strongly activated by various environmental stresses and inflammatory cytokines (40). Cellular Cell aging is referred to as stress-induced premature aging. Thus, the activation of JNK and p38 MAPK is closely associated with cell aging. In addition, in previous studies, when exposed to external harmful stimuli, p38 MAPK is majorly activated in Leydig cells (41–44), which may be due to cell type-specific effects. All these implied that p38 MAPK might be important regulator of Leydig cell aging.

Obesity is a well-recognized risk factor for cell aging (20). Clinical studies suggested that obesity could accelerate age-related decline in testosterone, but the underly mechanisms remain unclear. In our previous study, we demonstrated that obesity impaired testosterone synthesis in Leydig cells (29). In the present study, we found that the levels of p21 protein and the intensity of SA-β-Gal staining were higher in Leydig cells of obese mice, suggesting that obesity promoted Leydig cell premature aging. At the same time, we found that p38 MAPK was activated in Leydig cells of obese mice. This result indicated that p38 MAPK might be the key molecule linked obesity and Leydig cell aging.

To test these hypothesizes, a mouse model with p38 MAPK knockout in Leydig cells was established. In aged p38LCKO mice, Leydig cells underwent less senescence compared with aged WT mice. Accompanied by the remission of cell aging, the levels of testosterone and steroidogenic enzymes were increased. The resulted confirmed that reduced testosterone synthesis of aged Leydig cells was the consequence of cell aging, and p38 MAPK-mediated Leydig cell aging is an intrinsic mechanism of age-related decline in testosterone.

It has been reported that p38 MAPK may promote cell aging the following points. p38 MAPK facilitates the transcription of *p21* and *p16*, enhances the transcription of senescence-associated secretory phenotype (SASP) genes including IL-6, IL-8, and GM-CSF, and inhibits senescent cell apoptosis for pro-survival (45–48). In present study, the level of p21, not p16 protein was increased in aged Leydig cells. Meanwhile, KEGG analysis revealed p53 signaling pathway was activated in aged human Leydig cells. Based on these, we speculated that p38 MAPK promoted Leydig cell aging by p53/p21 signaling pathway. Future work could elucidate the detailed mechanisms.

It well known that both oxidative stress and inflammatory factors can activate p38 MAPK pathways to provoke cell adaptive response. Oxidative stress and inflammation are also the main factors inducing cell senescence. Aged Leydig cells produced significantly more reactive oxygen species (ROS) and had reduced expressions of key enzymatic and non-enzymatic antioxidants, leading cell oxidative stress (49–51). Our results showed that the levels of oxidative stress and pro-inflammatory factors were elevated in testis of aged mice, which suggested that inflammation and oxidative stress might be involved in the activation of p38 MAPK during Leydig cell aging.

Oxidative stress and inflammation are important mechanisms in the pathogenesis of obesity. In our previous studies, elevated levels of oxidative stress and inflammation in testis and Leydig cells of diet-induced obese mice (29, 52), suggesting that obesity promoted Leydig cell premature aging *via* enhanced oxidative stress and inflammation. The results from aged and obese mice implied that oxidative stress and inflammation are common factors of Leydig cell aging caused by age and obesity by activating the p38 MAPK pathway. As previously mentioned, obesity accelerates aging by shortening telomere length and increasing epigenetic aging and DNA damage (53–55). Whether obesity accelerates Leydig cell aging through the above mechanisms requires further research to confirm.

In generally, we demonstrated that p38 MAPK was involved in Leydig cell aging and aged-related decline in testosterone. Obesity was an important risk factor for aged-related decline in testosterone by promoting Leydig cell aging *via* activating p38 MAPK. The results provide a new insight to explore the mechanisms and look for intervention of age-related decline in testosterone.

## Data availability statement

The original contributions presented in the study are included in the article/**Supplementary Material**. Further inquiries can be directed to the corresponding authors.

## Ethics statement

The animal study was reviewed and approved by Animal Ethics Committee of Shandong Provincial Hospital.

## Author contributions

DL conducted most of the experiments and wrote the manuscript. XX analysed data. XQ and LY performed animal husbandry. CY and QG conceived of and designed the study. All authors contributed to the article and approved the submitted version.

## Supplementary material

Supplementary Figure 1 The protein levels of JNK, p-JNK, ERK1/2, and p-ERK1/2 in testis of 6- and 18-month-old mice were assessed by immunoblot **(A)**. Quantitative analysis for protein levels of p-JNK and p-ERK1/2 **(B, C)**.

Supplementary Figure 2 Assessment levels of proteins related to testosterone synthesis in mice fed with HFD or ND. The mRNA levels of these proteins were assessed by Q-PCR **(A)**. The protein levels of these proteins were Analyzed by immunoblot **(B)**. Quantitative analysis for these protein levels **(C)**.

Supplementary Figure 3 Assessment of ability of Leydig cell to produce testosterone in p38LCKO mice and WT mice. Testis morphology was examined by HE staining **(B)**. The mRNA levels of proteins and enzymes related to testosterone synthesis were assessed by Q-PCR **(C, E)**. The protein levels of proteins and enzymes associated with testosterone synthesis were Analyzed by immunoblot **(D, F)**.
