# Supplementary figures and images for: Involvement of p38 MAPK in Leydig cell aging and age-related decline in testosterone
Source: Front Endocrinol (Lausanne). 2023 Mar 6;14:1088249. doi: 10.3389/fendo.2023.1088249 (PMC10025507; doi:10.3389/fendo.2023.1088249)

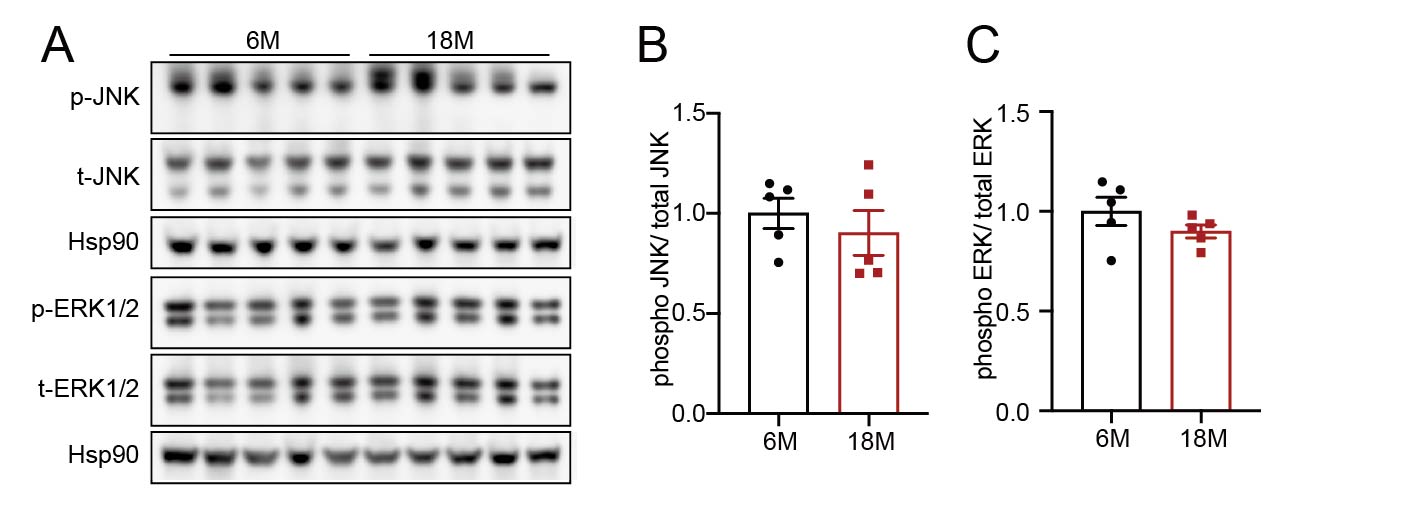

Supplement: Supplementary Figure 1 — The protein levels of JNK, p-JNK, ERK1/2, and p-ERK1/2 in testis of 6- and 18-month-old mice were assessed by immunoblot (A). Quantitative analysis for protein levels of p-JNK and p-ERK1/2 (B, C). [file Image_1.jpeg]

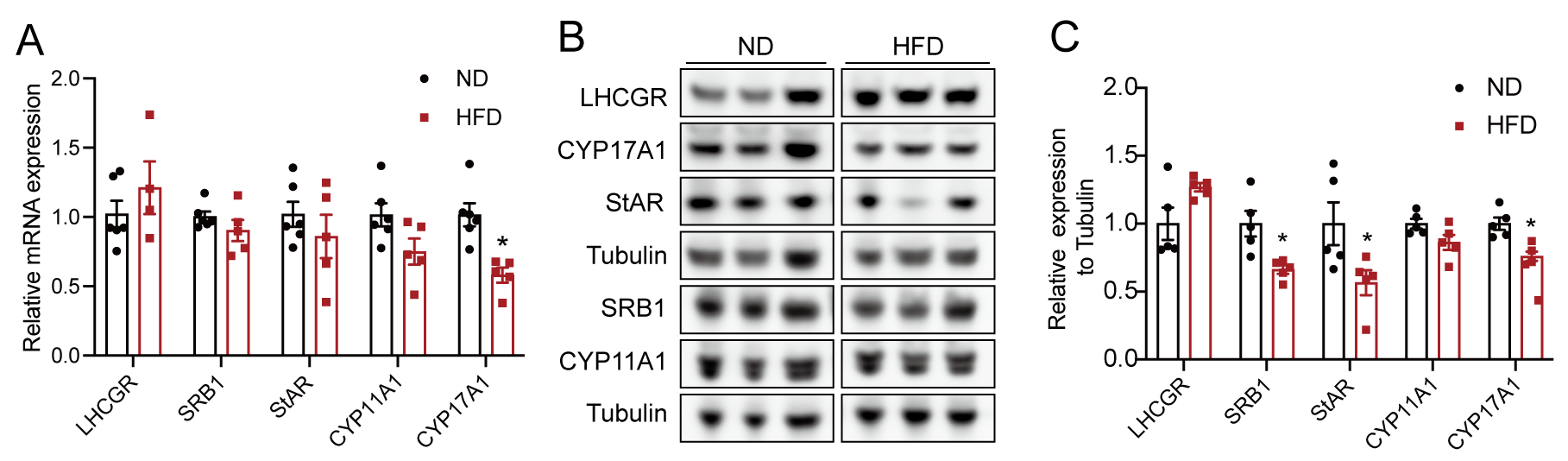

Supplement: Supplementary Figure 2 — Assessment levels of proteins related to testosterone synthesis in mice fed with HFD or ND. The mRNA levels of these proteins were assessed by Q-PCR (A). The protein levels of these proteins were Analyzed by immunoblot (B). Quantitative analysis for these protein levels (C). [file Image_2.tif]

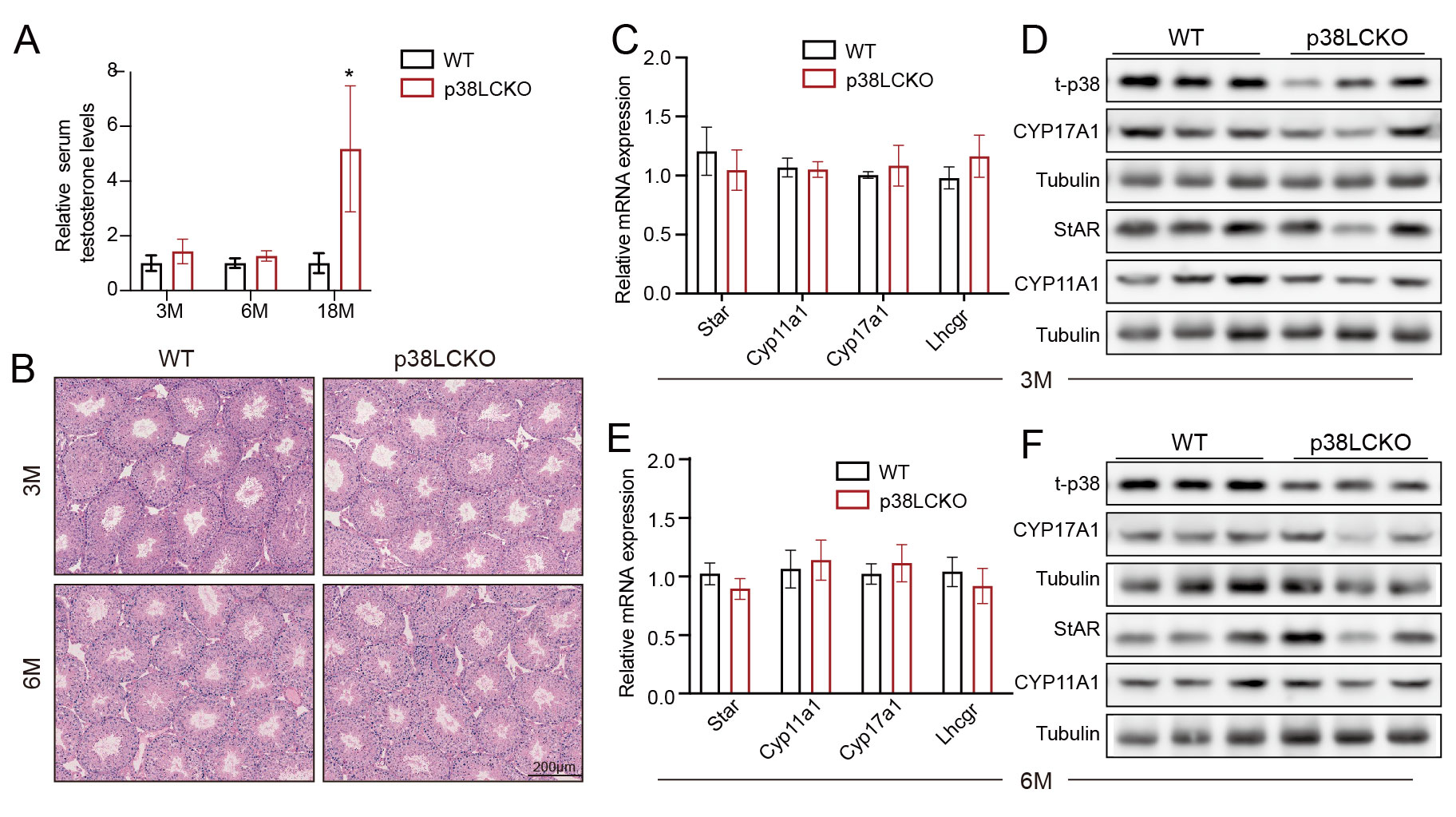

Supplement: Supplementary Figure 3 — Assessment of ability of Leydig cell to produce testosterone in p38LCKO mice and WT mice. Testis morphology was examined by HE staining (B). The mRNA levels of proteins and enzymes related to testosterone synthesis were assessed by Q-PCR (C, E). The protein levels of proteins and enzymes associated with testosterone synthesis were Analyzed by immunoblot (D, F). [file Image_3.jpeg]
